# Supplementary material for: Using a Battery of Bioassays to Assess the Toxicity of Wastewater Treatment Plant Effluents in Industrial Parks
Source: Toxics. 2023 Aug 14;11(8):702. doi: 10.3390/toxics11080702 (PMC10457805; doi:10.3390/toxics11080702)
Supplement: Supplementary file 1 [file toxics-11-00702-s001.zip › toxics-2505538-supplementary.pdf]

Supplementary Materials: Using a Battery of Bioassays to Assess the Toxicity of Wastewater Treatment Plant Effluents in Industrial Parks

Authors:

Bin Yang[1], Haiyan Cui[1], Jie Gao[1], Jing Cao[1], Göran Klobučar[2], Mei Li[1]*

Affiliations of authors:

[1] State Key Laboratory of Pollution Control and Resource Reuse, School of Environment, Nanjing University, Nanjing 210023, China

[2] Department of Biology, Faculty of Science, University of Zagreb, Rooseveltov trg 6, Zagreb, Croatia

***Corresponding author:**

Mei Li (Address: 163 Xianlin Ave., Nanjing University, Nanjing 210023, China)

Email address: meili@nju.edu.cn

Text S1 Health risk assessment through two exposure routes

The calculation formula of pollutant intake for each exposure route was as follows:

a. Route of oral exposure— $CDI_{\text{respiration}} = \frac{Cw \times ET \times EF \times ED \times (1/VF + 1/EF)}{BW \times AT}$ (1)

b. Route of skin exposure— $CDI_{\text{skin}} = \frac{Cw \times CF \times SA \times AF \times ABSd \times EF \times ED}{BW \times AT}$ (2)

In the formula, Chronic daily intake (CDI) is the sum of polycyclic aromatic hydrocarbons ingested through different exposure routes; Cw is the concentration of polycyclic aromatic hydrocarbons in the water (mg/L); VF is the volatility coefficient. The values of other parameters in the formula are shown in Table S1.

According to the research [1], when a specific exposure route lacks a toxicity parameter, it can be obtained through extrapolation:

$$RfD_{ABS} = RfD_0 \times ABS_{GI} \quad (3)$$

$$SF_{ABS} = SF_0 / ABS_{GI} \quad (4)$$

In the formula, RfD_{ABS} and RfD_0 are non-carcinogenic reference doses, SF_{ABS} and SF_0 are carcinogenic slope factors (kg·d/mg), ABS_{GI} is the polycyclic aromatic hydrocarbon absorption fraction and the conservative value is 0.5.

A. Non-carcinogenic risk index (HI)

For the non-carcinogenic risk assessment of eight polycyclic aromatic hydrocarbons, such as naphthalene, the calculation formula was as follows:

$$HI = \frac{CDI}{RfD} \quad (5)$$

B. Carcinogenic risk value (Risk)

The carcinogenic risk value was defined as: the incidence of cancer caused by exposure to this target pollutant exceeded the normal level in humans, which could be

calculated using the following formula:

Low-dose exposure— $Risk = CDI \times SF$ (6)

High-dose exposure— $Risk = 1 - \exp(-CDI \times SF)$ (7)

(when the carcinogenic risk value was greater than 0.01, we used this formula.)

Table S1 VF, RfD and CSF values of polycyclic aromatic hydrocarbons

Target pollutant	Volatile factor	Exposure route, non-carcinogenic Reference dose/(kg·d/mg)		Carcinogenic slope factor/(kg·d/mg)	
	VF	Skin (R_fDd)	Respiration (R_fDi)	Skin (SFd)	Respiration (SFi)
NAP	6.25×10^{4}	2.00×10^{-2}	8.57×10^{-4}	-	-
ACY	1.48×10^{5}	3.00×10^{-2}	3.00×10^{-2}	-	-
ACE	1.86×10^{5}	3.00×10^{-2}	3.00×10^{-2}	-	-
FLU	5.73×10^{5}	2.00×10^{-2}	2.00×10^{-2}	-	-
PHE	1.43×10^{6}	1.50×10^{-2}	1.50×10^{-2}	-	-
ANT	8.76×10^{5}	1.50×10^{-1}	1.50×10^{-1}	-	-
FLA	6.38×10^{5}	2.00×10^{-2}	2.00×10^{-2}	-	-
BaA	1.06×10^{7}	-	-	1.46	3.10×10^{-1}
BbF	5.24×10^{6}	-	-	1.46	3.10×10^{-1}
BaP	2.72×10^{7}	-	-	1.46×10^{1}	3.10
B（g,h,i）P	1.08×10^{8}	1.50×10^{-2}	1.50×10^{-2}	-	-

NAP: Naphthalene; ACY: Acenaphthylene; ACE: Acenaphthene; FLU: Fluorene; PHE: Phenanthrene; ANT: Anthracene; FLA: Fluoranthene; BaA: Benzo(a)anthracene; BbF: Benzo(b)fluoranthene; BaP: Benzo(a)pyrene; B(g,h,i) P: Benzo(g, h, i)perylene.

Table S2 Basic physical and chemical indicators of chemical parks

		pH	COD	AN	Ni	NN	SS	TOC	TN
W	Influents	9.54	5640	89.9	32.6	0.79	0.42	750	251
plant	Effluents	6.51	127	13.3	10.9	0.21	0.21	37.4	46.2
J	Influents	8.76	2901	79.2	30.7	0.94	0.57	426	205
plant	Effluents	7.21	112	15.4	6.3	0.13	0.26	33.5	37.9
T	Influents	8.37	4900	85.1	37.2	0.82	0.79	623	211
plant	Effluents	6.22	109	12.7	8.9	0.20	0.30	25.9	40.6

COD: chemical oxygen demand; AN: ammonia nitrogen (NH^{4+}-N); Ni: nitrate nitrogen (NO^{3-}-N); NN: nitrite nitrogen (NO^{2-}-N); SS: suspended solids; TOC: total organic carbon; TN: total nitrogen.

Table S3 Cytotoxicity of water extracts from the chemical parks to *S. typhimurium*

Plants	Wastewater type	Concentration factor	G value
W plant	Influent	0.5×	0.51
		1×	0.45*
		2×	0.25*
		5×	0.11*
	Effluent	0.5×	0.54
		1×	0.48*
		2×	0.47*
		5×	0.40*
J plant	Influent	0.5×	0.51
		1×	0.41*
		2×	0.40*
		5×	0.23*
	Effluent	0.5×	0.52
		1×	0.49*
		2×	0.48*
		5×	0.38*
T plant	Influent	0.5×	0.50
		1×	0.46*
		2×	0.31*
		5×	0.19*
	Effluent	0.5×	0.54
		1×	0.49*
		2×	0.48*
		5×	0.46*

G value: bacterial growth factor value. *, p <0.05, compared with the 0.5× group.

Table S4 Induction rate of the water extracts from the chemical industry parks on *S. typhimurium*

Plants	Wastewater type	Concentration factor	IR value
W plant	Influent	0.5×	2.09
		1×	-
		2×	-
		5×	-
	Effluent	0.5×	1.89
		1×	-
		2×	-
		5×	-
J plant	Influent	0.5×	2.13
		1×	-
		2×	-
		5×	-
	Effluent	0.5×	1.95
		1×	-
		2×	-
		5×	-
T plant	Influent	0.5×	2.15
		1×	-
		2×	-
		5×	-
	Effluent	0.5×	1.85
		1×	-
		2×	-
		5×	-

IR value: induction ratio value.

Table S5 Health risk index of non-carcinogenic effects of pollutants via different exposure routes according to the risk characterization model

	Respiratory exposure						Skin exposure					
Compound	W plant		J plant		T plant		W plant		J plant		T plant	
	Influent	Effluent	Influent	Effluent	Influent	Effluent	Influent	Effluent	Influent	Effluent	Influent	Effluent
NAP	1.55×10^{-5}	5.13×10^{-8}	1.77×10^{-5}	2.05×10^{-8}	1.44×10^{-5}	2.93×10^{-8}	1.17×10^{-5}	3.88×10^{-8}	1.33×10^{-5}	1.55×10^{-8}	1.09×10^{-5}	2.21×10^{-8}
ACY	2.89×10^{-8}	-	6.71×10^{-8}	4.90×10^{-10}	3.62×10^{-8}	-	6.74×10^{-8}	-	1.56×10^{-7}	1.14×10^{-9}	8.45×10^{-8}	-
ACE	3.79×10^{-9}	-	1.26×10^{-8}	3.18×10^{-10}	7.81×10^{-9}	1.59×10^{-10}	2.62×10^{-8}	-	8.75×10^{-8}	2.20×10^{-9}	5.40×10^{-8}	1.10×10^{-9}
FLU	-	1.78×10^{-8}	1.32×10^{-8}	-	1.25×10^{-8}	-	-	1.24×10^{-7}	9.22×10^{-8}	-	8.76×10^{-8}	-
PHE	3.12×10^{-9}	3.19×10^{-10}	4.98×10^{-9}	1.27×10^{-10}	-	-	5.43×10^{-8}	5.54×10^{-9}	8.65×10^{-8}	2.21×10^{-9}	-	-
ANT	1.28×10^{-7}	1.56×10^{-9}	1.47×10^{-7}	-	-	2.18×10^{-9}	1.36×10^{-6}	1.66×10^{-8}	1.57×10^{-6}	-	-	2.33×10^{-8}
FLA	2.23×10^{-10}	-	5.6×10^{-10}	3.86×10^{-11}	1.25×10^{-10}	-	2.85×10^{-8}	-	7.22×10^{-8}	4.94×10^{-9}	1.59×10^{-8}	-
BaA	3.71×10^{-10}	6.57×10^{-11}	1.49×10^{-9}	1.43×10^{-10}	1.85×10^{-10}	-	2.35×10^{-8}	4.18×10^{-9}	9.51×10^{-8}	9.13×10^{-9}	1.17×10^{-8}	-
BbF	1.19×10^{-9}	2.47×10^{-11}	1.48×10^{-9}	4.94×10^{-11}	1.38×10^{-9}	6.82E-11	3.86×10^{-7}	7.99×10^{-9}	4.79×10^{-7}	1.59×10^{-8}	4.46×10^{-7}	2.20×10^{-8}
BaP	7.89×10^{-8}	9.86×10^{-9}	2.09×10^{-7}	1.23×10^{-8}	1.66×10^{-7}	8.63×10^{-9}	1.41×10^{-7}	1.77×10^{-8}	3.76×10^{-7}	2.21×10^{-8}	2.99×10^{-7}	1.55×10^{-8}
B（g,h,i）P	3.21×10^{-9}	-	5.62×10^{-9}	7.14×10^{-11}	4.42×10^{-9}	-	4.97×10^{-8}	-	8.73×10^{-8}	1.10×10^{-9}	6.85×10^{-8}	-

NAP: Naphthalene; ACY: Acenaphthylene; ACE: Acenaphthene; FLU: Fluorene; PHE: Phenanthrene; ANT: Anthracene; FLA: Fluoranthene; BaA: Benzo(a)anthracene; BbF: Benzo(b)fluoranthene; BaP: Benzo(a)pyrene; B(g,h,i) P: Benzo(g, h, i)perylene; “-” : no health risks.

Table S6. Health risk index of carcinogenic effects of pollutants via different exposure routes according to the risk characterization model

	Respiratory exposure						Skin exposure					
Compound	W plant		J plant		T plant		W plant		J plant		T plant	
	Influent	Effluent	Influent	Effluent	Influent	Effluent	Influent	Effluent	Influent	Effluent	Influent	Effluent
NAP	4.52×10^{-5}	1.49×10^{-7}	5.16×10^{-5}	5.98×10^{-8}	4.22×10^{-5}	8.55×10^{-8}	3.42×10^{-5}	1.13×10^{-7}	3.90×10^{-5}	4.52×10^{-8}	3.19×10^{-5}	6.46×10^{-8}
ACY	8.43×10^{-8}	-	1.95×10^{-7}	1.42×10^{-9}	1.05×10^{-7}	-	1.96×10^{-7}	-	4.56×10^{-7}	3.33×10^{-9}	2.46×10^{-7}	-
ACE	1.10×10^{-8}	-	3.69×10^{-8}	9.29×10^{-10}	2.27×10^{-8}	4.64×10^{-10}	7.65×10^{-8}	-	2.55×10^{-7}	6.43×10^{-9}	1.57×10^{-7}	3.21×10^{-9}
FLU	-	5.18×10^{-8}	3.85×10^{-8}	-	3.64×10^{-8}	-	-	3.62×10^{-7}	2.69×10^{-7}	-	2.55×10^{-7}	-
PHE	9.12×10^{-9}	9.31×10^{-10}	1.45×10^{-8}	3.72×10^{-10}	-	-	1.58×10^{-7}	1.61×10^{-8}	2.52×10^{-7}	6.47×10^{-9}	-	-
ANT	3.73×10^{-7}	4.55×10^{-9}	4.31×10^{-7}	-	-	6.38×10^{-9}	3.98×10^{-6}	4.85×10^{-8}	4.59×10^{-6}	-	-	6.79×10^{-8}
FLA	6.51×10^{-10}	-	1.64	1.12×10^{-10}	3.64×10^{-10}	-	8.31×10^{-8}	-	2.10×10^{-7}	1.44×10^{-8}	4.65×10^{-8}	-
BaA	1.08×10^{-9}	1.91×10^{-10}	4.36×10^{-9}	4.18×10^{-10}	5.40×10^{-10}	-	6.88×10^{-8}	1.22×10^{-8}	2.77×10^{-7}	2.66×10^{-8}	3.44×10^{-8}	-
BbF	3.48E-09	7.20×10^{-11}	4.32×10^{-9}	1.44×10^{-10}	4.02×10^{-9}	1.99×10^{-10}	1.12×10^{-6}	2.33×10^{-8}	1.39E-06	4.66×10^{-8}	1.30×10^{-6}	6.43×10^{-8}
BaP	2.30×10^{-7}	2.87×10^{-8}	6.11×10^{-7}	3.59×10^{-8}	4.85×10^{-7}	2.51×10^{-8}	4.13×10^{-7}	5.18×10^{-8}	1.09×10^{-6}	6.44×10^{-8}	8.75×10^{-7}	4.55×10^{-8}
B（g,h,i）P	9.37×10^{-9}	-	1.64×10^{-8}	2.08×10^{-10}	1.29×10^{-8}	-	1.45×10^{-7}	-	2.54×10^{-7}	3.22×10^{-9}	2.00×10^{-7}	-

NAP: Naphthalene; ACY: Acenaphthylene; ACE: Acenaphthene; FLU: Fluorene; PHE: Phenanthrene; ANT: Anthracene; FLA: Fluoranthene; BaA: Benzo(a)anthracene; BbF: Benzo(b)fluoranthene; BaP: Benzo(a)pyrene; B(g,h,i) P: Benzo(g, h, i)perylene; "-" : no health risks.

Reference:

1. Feng H.-Y.; Fu X.-Q.; Zhao Q. Health risk assessment of polycyclic aromatic hydrocarbons in soils of Ningbo area, China. J Agro-Environ Sci. 2011 30(10), 1998-2004.
